# Personality traits, psychosocial effects and quality of life of patients submitted to dental bleaching

**DOI:** 10.1186/s12903-020-01370-6

**Published:** 2021-01-06

**Authors:** Elize Bonafé, Márcia Rezende, Marina Molinari Machado, Suellen Nogueira Linares Lima, Eduardo Fernandez, Marcia M. P. Baldani, Alessandra Reis, Alessandro Dourado Loguercio, Matheus Coelho Bandeca

**Affiliations:** 1 School of Dentistry, State University of Ponta Grossa, Ponta Grossa, PR 84030-900 Brazil; 2 School of Dentistry, School Paulo Picanço, Fortaleza, CE 60135-218 Brazil; 3 Postgraduate Program in Dentistry, Faculty of Dentistry, CEUMA University, Street Josue Montello s/n, São Luís, Maranhao 65075-120 Brazil; 4 Restorative Dentistry, University of Chile, Santiago, Chile; 5 Instituto de Ciencias Biomédicas, Universidad Autónoma de Chile, Providencia, Chile

**Keywords:** Personality, Psychosocial effect, NEO-FFI, Dental bleaching, Extraversion, Quality of life

## Abstract

**Background:**

Perception is defined as the ability to distinguish through the senses. All perception is dependent on factors such as personality, previously lived experiences and cultural elements. When planning an aesthetic treatment, consider the way the patients perceive the changes and outcomes is essential for reaching their expectations. The objective of this study was to assess if there was predominance of a personality trait of patient undergoing dental bleaching and if this treatment could promote changes in this traits, in the psychosocial impact and quality of life of these individuals.

**Methods:**

The assessment of personality characteristics, quality of life, psychosocial and self-perception was a cross-sectional observational study and it was carried out by applying questionnaires to 55 patients that were submitted to a clinical phase. The psychometric instruments used were NEO FFI-R (personality), PIDAQ (psychosocial effect) and WHOQOL-BREF (quality of life). Each test domain was prior and after bleaching by Wilcoxon Signed Rank test (α = 0.05). The internal consistencies of each scale were evaluated by Cronbach's alpha.

**Results:**

No statistical significant differences among personality traits means were observed among participants but there was predominance of two predominant personality traits in this study: conscientiousness (45.5%) and extraversion (34.5%). In four test domains of the PIDAQ, significant differences were observed before and after dental bleaching. The overall perception of the PIDAQ was also statistically significant demonstrating an improvement. There were no differences on overall or specific domains scores of the WHOQOL before and after treatment.

**Conclusions:**

Subjects who underwent dental treatment improved their self-confidence and reduced concerns about dental aesthetics, social and personality impact of dental alterations.

***Trial registration*:**

This study was conducted in parallel to a clinical investigation that aimed to evaluate tooth sensitivity related to dental bleaching technique and registered in REBEC clinical registry under protocol RBR-6pt2n3 in 13 November 2013.

## Background

Dentistry has been growing due to a significant increase in the demand for aesthetic treatments [1], especially dental bleaching [1–3]. However, the perception of beauty and aesthetics varies from person to person and among different cultures, highly influenced by personal experiences and psychological factors [4–6].

Some psychological traits could be directly correlated with negative body image (i.e., negative body image has been associated with higher levels of neuroticism and lower levels of extraversion) [6], and some people could be more satisfied with aesthetic treatments, while others could be more demanding [7–9]. In this context, personality is related to health disorder development and responses to treatment outcomes [10–15]. Therefore, it is important to know patients’ personality to improve oral care, understand their needs and intuit their demands. This topic has been very poorly explored in the literature in relation to dentistry.

Personality can be described as the dynamic organization of psychophysical systems that determine a person’s characteristics, behavior, thoughts and feelings [16]. Psychologists have discussed the meaningful changes in personality traits during adulthood for a long time [17]. These changes are complex and ongoing, due to many factors, such as social roles, life events, social environment [18, 19] and biological cases [20]. However, not all factors that can affect these traits are well known.

Currently, among the different analyses of personality structure, the model described as the “Big Five” [21] is the most popular [22]. According to this model, five traits associated with certain aspects represent the most important dimensions of personality [23]. These are extraversion, neuroticism, agreeableness, openness and conscientiousness. Unfortunately, studies that consider the personality profile of patients undergoing dental bleaching are scarce [15, 24]. However, recognizing the factors that could overestimate or underestimate the results of cosmetic treatments would be interesting in clinical planning and research [8].

Furthermore, including patients’ perceptions in planning the treatment and evaluating its outcome [5, 25–27] is crucial for meeting patients’ expectations. With this purpose, some questionnaires have been recommended, such as the PIDAQ (The Psychosocial Impact of Dental Aesthetics Questionnaire) [28]—which was originally developed to assess orthodontic patients [28] and was later used to evaluate other clinical conditions [29–31]—as well as WHOQOL-BREF (The World Health Organization Quality of Life—abbreviated version). Although several studies have evaluated the psychosocial profile and quality of life of patients undergoing dental bleaching [32–35], their application in in-office dental bleaching is scarce [3].

Therefore, taking in consideration that, tooth color has been cited as a major factor of dissatisfaction with the smile [1, 2], mainly in the young people and that, bleaching has become one of the cosmetic procedures that provide the greatest satisfaction to patients [1–3], the evaluation of personality traits, psychosocial impact and quality of life of patients undergoing dental bleaching is very important. Thus, the objective of this study was to assess if there is a predominant personality trait of patients undergoing in-office dental bleaching and if this treatment can promote change in some of the personality traits, psychosocial impact and quality of life of these individuals.

## Methods

This trial, nonrandomized, controlled before-and-after study was conducted in parallel to a clinical investigation that aimed to evaluate tooth sensitivity related to dental bleaching technique (REBEC clinical registry under protocol RBR-6pt2n3) on November 13, 2013. Both clinical studies, in the observational phase, were approved by the Scientific Review Committee and the Committee for the Protection of Human Participants of the local university (protocol numbers 172.988 and 1008.633, respectively). An exploratory longitudinal observational phase was designed encompassing the assessment of personality traits, overall health-related quality of life and the impact of dental esthetics on quality of life, and it was carried out by administering questionnaires to the group of patients participating in the clinical study previously described.

### Setting and location

The study was conducted from November 11, 2013, to March 3, 2014, in Ponta Grossa, a city in southern Brazil. The sample was composed to university students. All procedures were performed by two PhD students on patients who sought bleaching treatment at the School of Dentistry at the State University of Ponta Grossa. Recruitment was carried out by placing written ads on walls of the university, thus forming a sample of convenience.

### Eligibility criteria

Participants included in the clinical trial were between 18 and 35 years old. The participants were required to have central incisors of shade A1 or darker, judged by comparison with the value-oriented shade guide (Vita Lumin, Vita Zahnfabrik, Bad Säckingen, Germany). Some volunteers were excluded because they would not be suitable for a cosmetic procedure such as bleaching: participants with anterior restorations or dental prosthesis, with orthodontics apparatus and with severe internal tooth discoloration (tetracycline stains, fluorosis and pulpless teeth). In addition, pregnant and lactating women, participants with any other pathology that could cause sensitivity (such as recession, dentinal exposure and visible cracks in teeth), individuals taking anti-inflammatory or analgesic drugs, smokers, participants with bruxism, or participants who had undergone tooth-whitening procedures were also excluded.

Sixty-three patients were recruited for the follow-up observational phase of the study. This number of patients were calculated based on differences found in patients who completed the personality questionnaires [15] and psychosocial impact [3], considering a power of 0.8 and an alpha error of 0.05.

All participants signed an informed consent form, both for the completion of the bleaching itself and to permit information obtained through questionnaires to be used. All 63 patients were submitted to bleaching, but 8 patients did not complete the questionnaires and were excluded from this evaluation. Therefore, 55 patients (87.3%) completed all phases.

### Intervention: bleaching procedure

First, the gingival tissue of the teeth to be bleached (upper and lower maxillary six anterior teeth) was isolated using a light-cured resin dam (Top Dam, FGM, Joinville, SC, Brazil). Then, 35% hydrogen peroxide (HP) gel (Whiteness HP Maxx, FGM) was used in three 15-min applications according to the manufacturer’s directions. The in-office bleaching agent was refreshed every 15 min during the 45-min application period. Two sessions were performed, with a 1-week interval.

All questionnaires were administered before of procedure and 15 days after the second session of bleaching [36] to assess the personality traits (NEO FFI-R), psychosocial impact (PIDAQ) and the quality of life (WHOQOL-BREF).

### Personality traits assessment: NEO FFI-R

The NEO FFI-R questionnaire (Revised NEO Five-Factor Inventory) is a short self-report version of the NEO-PI-R (Revised NEO Personality Inventory) [22]. For this assessment, the Brazilian version [37], commercially available from Vetor (Vetor Editora Psico-Pedagógica Ltda, São Paulo, SP, Brazil) was used. It is a 60-item self-administered questionnaire that provides a brief and comprehensive measure of the five major domains (traits) of personality: neuroticism, extraversion, openness, agreeableness and conscientiousness.

The domains have 12 items each, evaluated using a five-point Likert scale (strongly disagree [SD] = 1, disagree [D] = 2, neutral [N] = 3, agree [A] = 4 and strongly agree [SA] = 5). The examiner encouraged patients to respond to all the items. The conscientiousness domain describes a careful, detail-oriented nature. A high score implies keeping things in order, coming prepared to school or work, being goal-driven and being persistent. A low score on conscientiousness might mean being less organized, completing tasks in a less structured way, taking things as they come, finishing things at the last minute and being impulsive. Agreeableness refers to a desire to keep things running smoothly. A high score on agreeableness might mean always being ready to help out, being caring and honest, being interested in the people around you and believing the best about others. A low agreeableness score might mean being stubborn, finding it difficult to forgive mistakes, being self-centered and having less compassion for others. Neuroticism describes a tendency to have unsettling thoughts and feelings. A high score on neuroticism can mean often feeling vulnerable or insecure, getting stressed easily, struggling with difficult situations and having mood swings. A low score on neuroticism indicates being likely to remain calm in stressful situations, being more optimistic, worrying less and having a more stable mood. Openness, or openness to experience, refers to a sense of curiosity about others and the world. A high score on openness means one might enjoy trying new things, be more creative, have a good imagination and be willing to consider new ideas. A low openness score might mean preferring to do things in a familiar way, avoiding change and being more traditional in your thinking. Extraversion refers to energy being drawn from social interactions. A high extraversion score might mean seeking excitement or adventure, making friends easily, speaking without thinking and enjoying being active with others. A low extraversion score can mean having a hard time making small talk or introducing yourself, feeling worn out after socializing, avoiding large groups and being more reserved.

Each patient received the booklet of items, the answer sheet and a pen. Patients had to answer each question by checking one of the response options on a Likert scale. There was no time limit for this test. In the cases of not understanding or doubt between responses, the respondents were instructed to mark the option “neutral.” For the evaluation of the NEO FFI-R (Vetor Editora, São Paulo, SP, Brazil), each response received a numerical value previously indicated by a blind operator, and the Riddle Computerized Correction on the computer, which is part of the whole questionnaire, was used. Numerical data were transcribed, and then a report was generated for each patient. The scores for each dimension were classified according to the NEO FFI-R standardization table. For the missing items, the mean of the other items of the same domain of each test was imputed [38, 39]; this simple imputation was standardized for all questionnaires applied.

### Quality of life assessment: WHOQOL-BREF

To assess quality of life, we used the WHOQOL-BREF (World Health Organization Qualify Of Life) questionnaire, which is a shortened version of the WHOQOL-100 instrument developed by the World Health Organization. The WHOQOL-BREF Brazilian version was developed and validated by Fleck et al. [40, 41]. It contains 24 questions in four domains: physical, psychological, social relationships and environment. There are also two additional questions that are intended to be examined separately: Question 1 asks about an individual’s overall perception of his or her QOL (quality of life), and question 2 asks about an individual’s overall perception of his or her own health. The items were rated on a 5-point Likert scale. For the first and second questions, the scale ranged from very poor = 1 to poor = 2, neither poor nor good = 3, good = 4 and very good = 5. For other questions, the scale ranged from not at all = 1 to a little = 2, a moderate amount = 3, very much = 4 and extremely = 5. Some items (3, 4 and 26) were recoded so that the values were inverted (5 = 1, 4 = 2, 3 = 3, 2 = 4 and 1 = 5). We followed the WHOQOL-BREF scoring guideline to score missing data in the questionnaire.

Domain facets incorporated within domains were as follows: (1) physical health related to activities of daily living, dependence on medicinal substances and medical aids, energy and fatigue, mobility, pain and discomfort, sleep and rest and work capacity; (2) psychological bodily factors related to image and appearance, negative or positive feelings, self-esteem, spirituality/religion/personal beliefs, thinking, learning, memory and concentration; (3) social relationships related to personal relationships, social support and/or sexual activity; and (4) environment, financial resources (related to freedom, physical safety and security), health and social care (accessibility and quality), home environment, opportunities for acquiring new information and skills, participation in and opportunities for recreation/leisure activities, physical environment (pollution/noise/traffic/climate) and/or transport.

### Psychosocial impact assessment: PIDAQ

The PIDAQ (The Psychosocial Impact of Dental Aesthetics Questionnaire), formulated by Klages et al. [28], is a specific questionnaire for assessing the psychosocial impact of dental aesthetics on young adults aged 18–30 years. We used the Brazilian validated version that has demonstrated satisfactory psychometric properties for Brazilian young adults [42]. It is a psychometric instrument composed of 23 items that uses negatively and positively worded items, divided into one positive and three negative domains, structurally composed of four subscales: “Aesthetic Concern” (3 items), “Psychological Impact” (6 items), “Social Impact” (8 items) and “Dental Self-Confidence” (6 items).

A five-point Likert scale is used, ranging from 0 (no impact of dental aesthetics on the psychosocial profile) to 4 (maximal impact of dental aesthetics on the psychosocial profile) for each item. The response options are 0 = not at all, 1 = a little, 2 = somewhat, 3 = strongly and 4 = very strongly. Each subscale score was calculated separately, and the score was obtained by summing the item scores. Where an item was missing, we substituted the mean of other items in the domain as the research criterion.

The PIDAQ measures three additional negative dimensions of psychosocial impact: social impact, psychological impact and aesthetic concern. Social impact aims to assess potential problems that an individual might face in social situations due to him or her having a subjectively unfavorable dental appearance. Psychological impact evaluates an individual’s feelings of inferiority or unhappiness compared with others. Aesthetics concern includes data pertaining to the concern or disapproval that an individual’s dental appearance generates when that individual looks in a mirror or views photographs or videos of him or herself.

### Statistical analysis

Data analysis was performed using SPSS 22.0 (SPSS Inc., Chicago, IL, USA and G*Power Version 3.0.10, Faul F, Universität Kiel, Germany). Descriptive analysis provided summary statistics of the demographic characteristics (Table 1). The internal consistencies of the tests were evaluated by alpha Cronbach's.

**Table 1:** Demographic characteristics of the sample.(p < 0.05)

Gender	
Male	(n = 23) 42.0%
Female	(n = 32) 58.0%
Age	
18–25	(n = 43) 79.0%
25–35	(n = 12) 21.0%
Educational level	
Primary education	0%
Complete secondary education	(n = 2) 3.6%
Undergraduate	(n = 50) 90.8%
Graduate	(n = 2) 3.6%
Post-graduate	(n = 1) 2.0%

To evaluate the test results for each domain of the NEO FFI-R, PIDAC and WHOQOL-BREF, we used the Wilcoxon signed-rank test (α = 0.05), comparing results prior to and after dental bleaching. For the analysis, we considered the mean and standard deviation, and we declared the minimum and maximum value, as well as the confidence intervals and the effect size achieved.

## Results

Fifty-five patients completed the questionnaires before and after the intervention [43]. All patients experienced an objectively measured color change of at least 7 units of delta E, considered effective tooth whitening.

Descriptive analysis provided summary statistics of the demographic characteristics (Table 1). The sample was composed mainly by volunteers between 18 and 25 years old who were undergraduate university students.

### Personality traits assessment: NEO FFI-R

After the intervention, no change in the personality traits of patients was observed (Table 2). However, there were two predominant personality traits in this study: conscientiousness (45.5%) and extraversion (34.5%). Neuroticism, openness and agreeableness showed medium scores. None of the 5 domains had significant changes related to bleaching procedure, not even trends.

**Table 2:** Means, standard deviations, minimum (min) and maximum (max) of responses for each domain of the NEO FFI-R questionnaire (*)

Domain	Mean ± SD		Min (before/after)	Max (before/after)	*p* value (*)	Effect size
	Before	After				
Neuroticism	24.3 ± 4.3	24.5 ± 4.7	16/16	35/35	0.88	0.02
Extroversion	28.9 ± 3.4	29.4 ± 3.3	21/20	35/41	0.40	0.07
Openness	25.3 ± 3.0	25.7 ± 2.7	18/21	37/34	0.18	0.22
Agreeableness	23.3 ± 4.0	23.6 ± 4.4	13/14	34/31	0.62	0.19
Conscientiousness	29.6 ± 4.0	30.0 ± 3.6	21/22	38/39	0.29	0.05

*Different periods were compared with the Wilcoxon Signed Rank (α = 0.05); Cronbach’s Alpha: 0.58

### Aesthetic self-perception assessment: PIDAQ

According to the PIDAQ results, significant improvement could be observed in aesthetics self-perception after dental treatment, in overall perception and in each of the four domains of the PIDAQ (Table 3). There was a significant increase in score for dental confidence, and a significant decrease for social impact, psychological impact and aesthetic concern. In summary, there was an improvement in all the factors of the psychosocial impact following the intervention. The internal consistency of the scale was high (Cronbach´s alpha ranging from 0.62 for psychological impact domains to 0.80 for esthetic concern domain).

**Table 3:** Means, standard deviations, minimum (min) and maximum (max) of responses for each domain of the PIDAQ questionnaire as well as the *p-value* for each domain and overall perception (*)

Domains	Mean ± SD		Min (before/after)	max (before/after)	*p* value (*)	effect size	Statistical power post hoc
	Before	After					
Dental self confidence	10.9 ± 4.8	16.2 ± 5.0	0/4	20/24	< 0.0001	0.47	0.99
Social impact	4.8 ± 4.7	3.4 ± 3.8	0/0	20/16	0.02	0.16	0.95
Psychological impact	6.5 ± 4.7	2.9 ± 2.6	0/0	15/9	< 0.001	0.42	0.95
Aesthetic concern	2.7 ± 2.7	2.0 ± 2.2	0/0	12/9	0.03	0.14	0.70

*Different periods were compared with the Wilcoxon Signed Rank (α = 0.05)

### Quality of life assessment: WHOQOL-BREF

With this instrument, an increase in values for each domain item and the overall evaluation of quality of life represents an improvement. There were no differences in the overall and specific domain scores of the WHOQOL before and after dental bleaching (Table 4). Scores for all domains were initially medium–high and were not significantly modified by the intervention. The initial quality of life level of this sample was good and did not change, according to this questionnaire.

**Table 4:** Means, standard deviations, minimum (min) and maximum (max) of responses for each domain of the WHOQOL-BREF questionnaire as well as the *p-value* for each domain and overall score (*)

Domains	Mean ± SD		Min (before/after)	Max (before/after)	*p* value (*)	Effect size
	Before	After				
Physical	13.0 ± 1.8	12.8 ± 1.5	17/15	29/29	0.20	0.06
Psychological	14.3 ± 1.4	14.4 ± 1.4	17/17	27/29	0.42	0.03
Social relationships	15.8 ± 2.2	15.4 ± 2.3	7/7	15/15	0.15	0.08
Environment	13.5 ± 1.8	14.1 ± 2.0	17/23	37/51	0.06	0.15
Overall perception of quality of life	15.8 ± 2.0	16.2 ± 1.4	75/75	122/121	0.06	0.11

*Different periods were compared with the Wilcoxon Signed Rank (α = 0.05)

Cronbach’s Alpha: 0.825

## Discussion

This study assessed the alterations in psychosocial impact of patients submitted to dental bleaching. In general, the results demonstrated that bleaching improved cosmetic dental confidence and decreased aesthetic concern, psychological impact and social impact. Some studies [42, 44, 45] demonstrated that the psychosocial impact of dental aesthetics is correlated with the severity of malocclusions [46, 47], with special regard to factors such as increased overjet, tooth displacement and increased overbite. Thus, it has the tendency to improve when orthodontic treatment is carried out [48, 49]. To the best of our knowledge, this is the first study that assessed this impact on patients submitted to a dental aesthetics treatment.

Although our study was not concerned with any changes in shape or tooth position, there was a perception of overall improvement, suggesting that changes in tooth color can improve patients’ satisfaction with their smile, even in the absence of other changes.

In fact, tooth color has been cited as a major factor of dissatisfaction with the smile [1, 2, 50, 51], and subjects who are quite satisfied with their smile are more likely to show their teeth and even to look at their teeth in the mirror [52]. It has even been argued that dental bleaching might be associated with increased self-esteem, and that this additional confidence could be associated with behavioral changes, resulting in changes interpersonal relationships [53]. Moreover, bleaching has become one of the cosmetic procedures that provide the greatest satisfaction to patients [2, 54].

Dental implants, orthodontic treatment and periodontal therapy have been associated with improved quality of life in dentistry [10, 11, 14], but our results suggest that dental bleaching did not affect the participants’ quality of life. Studies related to how bleaching influences quality of life are controversial, with some showing that dental bleaching improves quality of life [32] and others agreeing with the present study, showing that dental bleaching has no impact on patients’ quality of life [3, 33, 34].

This difference can be attributed to the different instruments used for the evaluation of quality of life (specific and general instruments). General instruments can be used for patients regardless of disease or condition, as well as for healthy people. They provide comparisons of common disease sufferers, different diseases or the general population [55]. However, they might fail in sensitivity to particular aspects. Specific instruments can detect particularities of quality of life in certain circumstances, such as diseases or treatments, providing relevant information for the management of patients [56]. According to Orley et al. [57], if the objective is to evaluate the influence of symptoms on the quality of life, it is recommended to use generic instruments in the assessment, but, unfortunately, we could not verify differences using only the WHOQOL, which suggests that a specific instrument could detect different results. Given the difficulty of evaluating specific situations with psychometric instruments, recently, another approach to analyze oral health quality of life is a synthesis of questionnaires, a combined instrument [58], which could be a valid strategy in future studies.

However, other methodological differences could also explain these controversial results. Apart from the different questionnaires being applied, the measurement of quality of life is very subjective and dependent on the population evaluated. For instance, a closer analysis of the cited studies [32–34] also showed a significant difference between population sample ages. Furthermore, the bleaching products and protocols applied could be responsible for the different results. McGrath et al. [34] evaluated several commercially available tooth-whitening products (toothpaste, adhesive strips and paint-on gel containing varying concentrations of hydrogen peroxide) without any restriction. Bruhn et al. [33] applied strips with 14% hydrogen peroxide, and Meirelles et al. [32] used 10 and 16% of carbamide peroxide applied in a tray-delivered system.

To the extent of our knowledge, this is the first study to evaluate the quality of life of patients submitted to in-office bleaching. All these differences prevent us from coming to a clear conclusion about the impact of dental bleaching on participants’ quality of life. Future studies need to be conducted comparing the quality of life before and after different bleaching therapies, in several populations and with different age groups.

Patient-reported outcomes are becoming important due to the relevance of patients’ well-being and health care [59]. There is some evidence that patients who receive what they expect are likely to recover better than patients who do not [60]. Some factors, such as personality traits, have been considered predictors for general well-being [61, 62] and seem to have a significant impact on individual satisfaction with therapies. Among instruments used to assess personality traits, the NEO-FFI has long been used, as it presents a strong scientific foundation [63]. This questionnaire facilitates the understanding of the personality of individuals and groups [22].

No differences in personality traits were observed through the NEO-FFI. This was expected, because personality traits are not correlated with specific behaviors or the sum of them but are global and abstract dispositions that summarize trends, styles and individual preferences [23]. These results are in agreement with a recent paper published by Herrera et al. [15], which showed no change in the scores on the 5 personality factors before and after at-home bleaching. Personality does not change based on immediate interventions, but remains rather stable over time.

On the other hand, the NEO-FFI demonstrated that there was a predominance of people with the conscientiousness and extroversion personality traits, which agrees with previous findings from Herrera et al. [15]. The prevalence of these two traits is somewhat different from that found by McCrae and Terraciano [64]. In their multicultural study, they verified a higher prevalence of two traits in the studied Brazilian population: neuroticism and extroversion. This difference in our sample in comparison with the Brazilian population could be related to the fact that, in our study, a nonrandom sample was obtained. In a nonrandom sample, some members of the population might not have any chance of being selected, which could be considered a limitation of the present study. Furthermore, we need to consider that, although the results of the present study showed significant differences for some personality traits, the prevalence rates in the present study could have a likely error of estimation due to the application of a small sample size when compared with the population prevalence. Future studies should focus on evaluation of the personality traits of patients submitted to in-office bleaching with a large randomized sample.

However, our results are also consistent with a study by Martin et al. [24], who assessed personality using the Millon questionnaire and found a correlation of patients seeking whitening with higher scores on the extroversion factor, similar to the results of this study. Despite of the some cultural differences among the populations evaluated in the mentioned studies [15, 24, 64], all of them strengthen the idea of patients submitted to dental bleaching score higher on the extroversion factor. Taking in consideration that the interculture differences, could be influenced by personal experiences and psychological factors [4–6], future studies that compare these effects (i.e. multicultural levels) in different populations and patients submitted to dental bleaching therapies (at-home or in-office) would be interesting.

It is important to note that another limitation of this research was that the population studied was young; in elderly people, there might be other predominant personality traits. It is known, for example, that with aging, there is a tendency for traits of neuroticism, extroversion and opening to diminish and for agreeableness and conscientiousness to increase [22]. In the same way, the aesthetic perception of elderly individuals cannot coincide with youths’ perceptions and needs. Perhaps a more heterogeneous sample in terms of age range or multicultural level could have been relevant in finding other results, especially in the WHO Qol Brief questionnaire, which started with high scores in this sample and would hardly vary with teeth whitening.

Finally, it is worth mentioning that the findings of the present study might be in part the effect of the people characteristics of undergoing bleaching treatment. A recent paper published by Herrera et al. [15], who studied a sample of a Chilean population, showed differences between people who underwent dental bleaching and people who refused it [15]. The subjects that effectively participated in that study were more extroverted. Although extroverts are more accepting of aesthetic therapies, their inherent optimism also enables better acceptance of the results. However, we envision several limitations due to being a pioneering study in this area. Several comparisons have limited statistical power, which makes validity difficult, but this study will help future researchers to better delineate their research. Perhaps the biggest extrapolation of this study is that the volunteers experienced a positive psychosocial effect, with a treatment considered to involve minimal intervention, which is relevant.

Unfortunately, these changes might not be sustained over a prolonged period, mainly because one of the most important concerns related to in-office bleaching is that the color could change in a few months. Matis et al. [65] showed that although there was a significant whitening effect immediately after bleaching, a color reversal in an order of 51% and 65% occurred after 1 and 6 weeks post-bleaching, respectively, for eight in-office products clinically evaluated. This means that all the changes observed in the present study might not be sustained over a prolonged period. Therefore, future studies need to be conducted evaluating the psychosocial impact and quality of life of patients undergoing dental bleaching after a long period.

## Conclusion

In conclusion, the results suggest that subjects who undergo dental bleaching treatment can improve in their confidence in dental appearance and reduce concerns about dental aesthetics and the social and psychological impact of dental alterations.

## Data Availability

The data and materials are available in the following repository: https://tede2.uepg.br/jspui/handle/prefix/2594.

## Abbreviations

NEO FFI-R: NEO Five-Factor Inventory
PIDAQ: The Psychosocial Impact of Dental Aesthetics Questionnaire
WHOQOL-BREF: The World Health Organization Quality of Life—abbreviated version
NEO-PI-R: NEO Personality Inventory
SD: Strongly disagree
D: Disagree
N: Neutral
A: Agree
SA: Strongly agree
QOL: Quality of life
